# Ferrofluid Microdroplet Splitting for Population‐Based Microfluidics and Interfacial Tensiometry

**DOI:** 10.1002/advs.202000359

**Published:** 2020-06-09

**Authors:** Mika Latikka, Matilda Backholm, Avijit Baidya, Alberto Ballesio, Amandine Serve, Grégory Beaune, Jaakko V. I. Timonen, Thalappil Pradeep, Robin H. A. Ras

**Affiliations:** ^1^ Department of Applied Physics Aalto University School of Science Puumiehenkuja 2 Espoo 02150 Finland; ^2^ Department of Chemistry Indian Institute of Technology Madras Chennai 600036 India; ^3^ Department of Bioproducts and Biosystems Aalto University School of Chemical Engineering Kemistintie 1 Espoo 02150 Finland

**Keywords:** ferrofluids, fluid dynamics, interfacial tension, magnetic fields, magnetic nanoparticles, microfluidics

## Abstract

Ferrofluids exhibit a unique combination of liquid properties and strong magnetic response, which leads to a rich variety of interesting functional properties. Here, the magnetic‐field‐induced splitting of ferrofluid droplets immersed in an immiscible liquid is presented, and related fascinating dynamics and applications are discussed. A magnetic field created by a permanent magnet induces instability on a mother droplet, which divides into two daughter droplets in less than 0.1 s. During the splitting process, the droplet undergoes a Plateau–Rayleigh‐like instability, which is investigated using high‐speed imaging. The dynamics of the resulting satellite droplet formation is shown to depend on the roughness of the supporting surface. Further increasing the field results in additional splitting events and self‐assembly of microdroplet populations, which can be magnetically actuated. The effects of magnetization and interfacial tension are systematically investigated by varying magnetic nanoparticles and surfactant concentrations, and a variety of outcomes from labyrinthine patterns to discrete droplets are observed. As the splitting process depends on interfacial tension, the droplet splitting can be used as a measure for interfacial tension as low as 0.1 mN m^−1^. Finally, a population‐based digital microfluidics concept based on the self‐assembled microdroplets is presented.

## Graphical abstract

Magnetic‐field‐induced instability triggers a splitting of a ferrofluid droplet immersed in immiscible liquid, creating two daughter droplets and several tiny satellite droplets. Further increasing the field results in additional splitting events, creating a self‐assembled microdroplet population, which can be used in population‐based digital microfluidics. This phenomenon can also be used to measure interfacial tension in liquid–liquid systems.

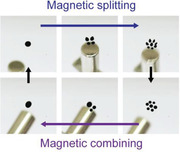

Ferrofluids are remarkably controllable materials allowing for magnetic manipulation of their shape, viscosity, flow, and heat transfer properties.^[^ ^1^, ^2^, ^3^, ^4^, ^5^ ^]^ Also magnetic properties can be tuned; a ferrofluid droplet can be reversibly switched from superparamagnetic to ferromagnetic by interfacial jamming of nanoparticles.^[^ ^6^ ^]^ Due to their versatility, ferrofluids find use in a range of applications from simple magnetically retained seals and lubricants to microfluidics and biomedical devices.^[^ ^2^, ^7^, ^8^, ^9^, ^10^, ^11^, ^12^, ^13^ ^]^ Recently, they have shown potential as multifunctional^[^ ^14^ ^]^ and anti‐icing surfaces,^[^ ^15^ ^]^ wearable sensors,^[^ ^16^ ^]^ probes for wetting characterization,^[^ ^17^ ^]^ and even liquid robotics.^[^ ^18^, ^19^ ^]^ Ferrofluids can also undergo fascinating ferrohydrodynamic instabilities, where a small variation of a control parameter (such as external magnetic field) causes an abrupt change in the ferrofluid configuration.^[^ ^1^ ^]^ These are interesting from a physics point of view, but also useful for applications, such as ferrofluid molding^[^ ^20^ ^]^ and field‐induced mixing in microfluidics.^[^ ^21^ ^]^ Here we present the magnetic‐field‐induced instability leading to splitting and self‐assembly of ferrofluid microdroplets immersed in immiscible liquid (**Figure** **1**a) as well as related potential applications. The ferrofluid we use is a colloidal suspension of citrate‐stabilized magnetite nanoparticles in water (synthesis procedure and in‐depth analysis are presented in ref. [22]). The aqueous ferrofluid droplet is placed in an immiscible solvent (e.g., octane or silicone oil) and subjected to an increasing magnetic field created by a permanent magnet underneath (see the text and Figure S1 in the Supporting Information for details). At a critical field strength and gradient, the droplet becomes unstable and splits into two daughter droplets, which has not been previously shown for ferrofluid droplets immersed in another liquid. The splitting process gives rise to another instability; as the ferrofluid bridge connecting the two daughter droplets gets thinner, it breaks up into satellite droplets with orders of magnitude smaller volumes than the daughter droplets. This phenomenon is similar to Plateau–Rayleigh instability, which leads to breakup of a falling liquid stream.^[^ ^23^ ^]^ We investigated the satellite droplet formation in detail using high‐speed imaging and found that the dynamics depends on the roughness of the supporting substrate. Numerous subsequent splitting events can be triggered by increasing the external field further after the first splitting, creating multiple generations of daughter droplets, which self‐assemble into well‐defined mobile patterns as guided by their mutual magnetic repulsion and attraction toward the permanent magnet. The shape and number of droplets depend on the ferrofluid magnetization and interfacial tension (IFT), which we varied by controlling the volume percentage of the superparamagnetic iron oxide nanoparticles (SPIONs), surfactant type, and surfactant concentration. As the field‐induced instability is governed by the IFT in addition to the magnetic field, the size of the split droplets can be used to determine IFTs as low as 0.1 mN m^−1^ with a simple theoretical model, well below the sensitivity limit of the commonly used pendant drop method. Finally, we demonstrate how the self‐assembled droplet patterns can be magnetically actuated and used in population‐based digital microfluidics, which allows switching between population‐level and droplet‐level controls.

**Figure 1:**
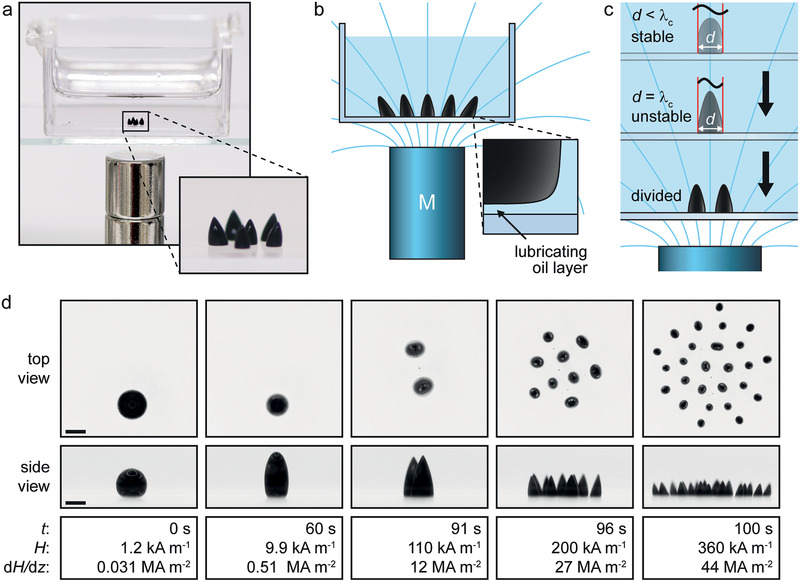
Magnetic‐field‐induced ferrofluid droplet splitting in an immiscible liquid. a) Photo of ferrofluid droplets in a polystyrene container filled with silicone oil and a stack of two cylindrical magnets (diameter and height = 9.5 mm) underneath. b) Schematic of a droplet population in a magnetic field (field lines in cyan) created by a permanent magnet. Inset shows a lubricating oil layer between the droplet and the substrate. c) Schematic of droplet splitting in an increasing magnetic field (*λ*_c_: critical wavelength, *d*: droplet diameter). d) Top and side views of ferrofluid droplet splitting in silicone oil (*t*: time, *H*: external magnetic field, and d*H*/d*z*: vertical field gradient). The distance between the magnet (diameter = 20 mm, height = 42 mm) and the droplets is reduced from 102.8 to 2.8 mm at a speed of 1 mm s^−1^. Scale bar: 1 mm.

The shape of a ferrofluid droplet is determined by magnetic, gravitational, and interfacial tension forces (when forces related to wetting are assumed negligible).^[^ ^24^, ^25^, ^26^ ^]^ The interfacial free energy is minimized when the droplet is spherical, while gravitational and magnetic forces deform this shape. Ferrofluid magnetization elongates the droplet along the field direction, and the strength of this effect compared to interfacial tension can be quantified with a dimensionless parameter $S=\mu_{0}M^{2}V^{\frac{1}{3}}\sigma^{-1}$, where *μ*_0_ is the vacuum permeability, *M* is the magnetization of the ferrofluid, *V* is the volume of the droplet, and *σ* is the interfacial tension between ferrofluid and surrounding fluid.^[^ ^27^ ^]^ In our work, we use a permanent magnet underneath the ferrofluid, which creates a nonuniform magnetic field affecting the droplets with a vertical magnetic force density $f_{M}=\mu_{0}\left(\bar{M}\cdot \nabla \right)\;\bar{H}=\mu_{0}\;MdH/dz$,^[^ ^1^ ^]^ where *H* is the external magnetic field. We approximate *M* and vertical field gradient d*H*/d*z* as constant over droplet volume, calculated at the center of the droplet. Together with the gravitational force density *f*_G_ =  Δ*ρ* *g* (Δ*ρ* is the density difference between ferrofluid and the surrounding fluid, and *g* is the gravitational acceleration) the normal force density *f*_N_ = *f*_G_  +*f*_M_ pulls the droplet against the substrate, flattening it. This can be quantified relative to the interfacial tension using the effective Bond number $B_{e}=f_{N}\;V^{\frac{2}{3}}\sigma^{-1}$.^[^ ^27^ ^]^

The interplay between interfacial, gravitational, and magnetic forces gives rise to interesting phenomena, including field‐induced instabilities. A classic example is the Rosensweig instability, where a uniform vertical magnetic field creates a macroscopic array of spikes on a horizontal ferrofluid surface.^[^ ^1^ ^]^ The periodicity of the array is determined by the critical wavelength $\lambda_{c}^{\mathrm{Rosensweig}}=\;2\pi \sqrt{\sigma /f_{G}}$. In case of a nonuniform magnetic field created by a permanent magnet, the critical wavelength can be written analogously as^[^ ^24^ ^]^

(1) $$\lambda_{c}=\;2\pi \sqrt{\frac{\sigma }{f_{N}}}$$

If the ferrofluid volume is small, the spikes can continue all the way down to the substrate. On a sufficiently liquid‐repellent surface, there is no ferrofluid film connecting the spikes, and individual droplets are created instead. This was first experimentally demonstrated by using superhydrophobic surfaces, where a thin air layer separates the droplet from the substrate (Cassie state of wetting) allowing droplets to move with little friction.^[^ ^24^ ^]^ Contrary to the previous experiments done in air,^[^ ^24^, ^26^, ^28^ ^]^ we achieve here repellency by immersing the ferrofluid droplets in an immiscible liquid, which creates a lubricating liquid film between the droplets and the substrate (Figure 1b; Figure S2, Supporting Information).^[^ ^29^, ^30^ ^]^ When the ferrofluid droplet diameter *d* is smaller than *λ*_c_, the droplet remains stable and is only deformed by the field (Figure 1c, top image). However, when *d* = *λ*_c_, the droplet becomes unstable and splits into two daughter droplets (Figure 1c, bottom image). A comprehensive theoretical discussion about the phenomena has recently been presented by Vieu and Walter.^[^ ^26^ ^]^ The splitting event takes less than 0.1 s, depending on the viscosities of the ferrofluid and the surrounding liquid. The split droplets are magnetized by the external field and are attracted to the global field maximum at the magnet's axis. Since the droplets are magnetized in the same direction, there is also dipolar interdroplet repulsion, leading to a symmetric, self‐assembled droplet pattern.^[^ ^24^ ^]^

As can be seen in the top view images of a splitting experiment (Figure 1d; Movie S1,Supporting Information), small satellite droplets are formed between the daughter droplets during the splitting event (**Figure** **2**a). To study the formation dynamics of these satellite droplets, we performed high‐speed imaging of a single splitting event at high spatial resolution (Figure 2b) in a polystyrene (PS) container filled with 5 cSt silicone oil. A rectangular magnet was used to induce splitting along the direction of the long side of the magnet, which allowed for side‐view imaging where both daughter droplets and all satellite droplets remained in focus during the entire splitting event.

**Figure 2:**
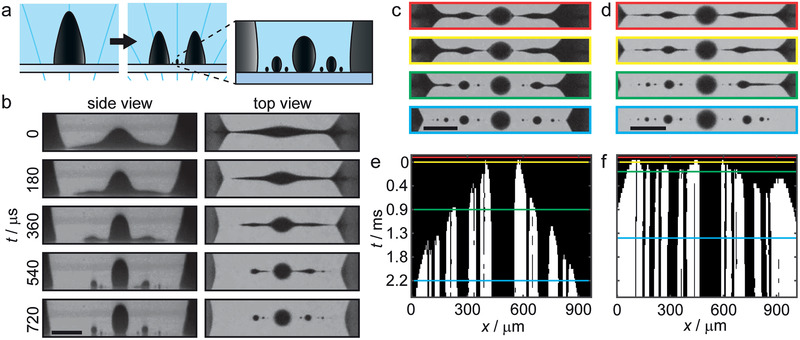
Dynamics of satellite droplet formation. a) Schematic of a mother droplet splitting into two daughter droplets. The zoomed inset shows small satellite and subsatellite droplets, which are formed during the splitting process due to the Plateau–Rayleigh‐like instability. b) Side‐ and top‐view snapshots of the breakup of the capillary bridge between two splitting daughter droplets on the Glaco‐coated substrate (SPION concentration 22 vol%, Movie S2, Supporting Information). The time between each picture is 180 µs. c,d) Top‐view snapshots of the capillary bridge breakup on the PS and Glaco surfaces, respectively (SPION concentration 24 vol%; see the colored lines in the graphs below for time stamp information). e,f) Corresponding graphs showing the detailed time evolution of the bridge breakup (black corresponds to the ferrofluid and white the surrounding oil). Before *t* = 0 ms, the bridge is still intact (red lines and boxes) and at *t* = 0 ms, the first pinch‐off occurs (yellow lines and boxes). On the smooth PS surface, the breakup starts around the largest satellite droplet in the center and continues outward in a symmetric manner (green and cyan lines and boxes). The entire breakup event takes several milliseconds. In comparison, on the rough Glaco‐coated surface, the breakup starts almost simultaneously at the center and the edge of the bridge, and evolves then quickly inward from both sides in a total breakup time of 0.5 ms. All scale bars: 0.2 mm.

Toward the end of each splitting event, a capillary bridge is formed between the two daughter droplets (Figure 2b). This capillary bridge undergoes an interfacial‐tension‐driven Plateau–Rayleigh‐like instability,^[^ ^31^, ^32^, ^33^ ^]^ where a disturbance of a specific wavelength is amplified, leading to the breakup of the bridge. The creation of satellite droplets during the breakup process is a highly nonlinear phenomenon and has been carefully studied by Tjahjadi et al.,^[^ ^23^ ^]^ combining experiments with boundary‐integral calculations to investigate the time evolution of a capillary oil bridge suspended in corn syrup. In our system, however, the liquid bridge consists of non‐Newtonian magnetic fluid, whose viscosity changes with the external magnetic field, thus requiring complicated magnetohydrodynamics for it to be fully modeled.^[^ ^2^ ^]^ Furthermore, our ferrofluid droplets are substrate‐supported, and the added drag effect can influence the fluid dynamics of the satellite droplet formation. For these reasons, we made a purely qualitative investigation on the satellite droplet formation using two substrates of different roughness (Figure S3a,b, Supporting Information): a smooth PS surface (RMS roughness = 6.2 ± 0.2 nm), and a PS surface coated with the commercial superhydrophobic Glaco coating (RMS roughness = 43 ± 3 nm).

In Figure 2b, the satellite droplet formation process on the Glaco‐coated surface is shown as time‐lapse images from the side and from the top (Movie S2, Supporting Information). The top view shows the ferrofluid bridge breaking up in a repeated, self‐similar fashion into a single large satellite droplet surrounded by ≈10 tiny subsatellite droplets. The resulting droplet size distribution is fractal like, similar to a droplet population resulting from a breakup of a Newtonian capillary bridge in a viscous, infinite medium.^[^ ^23^ ^]^ From the side view, however, our magnetic system is very different since the height of the bridge is affected by the external magnetic field, rendering ellipsoidal rather than spherical satellite droplets. Within the spatial sensitivity of our experiments, we find no clear difference between the two substrates in the final number, size, or spacing between the satellite droplets (Figure 2c,d). However, a strong effect is seen in the dynamics of the breakup and satellite droplet formation on the PS and Glaco‐coated substrates (Figure 2e,f). The fractal‐like time evolution graphs show the breakup as viewed along the horizontal symmetry axis, where black corresponds to the ferrofluid and white shows the surrounding media. On the PS substrate, the breakup starts at the center and moves symmetrically outward as a function of time, while on the Glaco‐coated substrate the breakup starts almost simultaneously at the center and the edge of the ferrofuid bridge and continues at a much faster pace than on the PS substrate. We hypothesize the greater roughness of the Glaco‐coated surface allows increased oil flow between the surface asperities compared to the smooth PS surface (Figure S2b,c, Supporting Information). This reduces shear stress in the lubricating layer and enhances droplet mobility, leading to faster splitting dynamics. Supporting Information contains more detailed discussion on the experiments and the effect of ferrofluid density on the time‐evolution dynamics on the two different substrates (Figure S3c,d, Supporting Information).

Further increasing the magnetic field after the first droplet splitting leads to numerous sequential splitting events, which were investigated using a cylindrical permanent magnet to create radially symmetric self‐assembled droplet populations. Here we focused on the self‐assembly of the daughter droplets and ignored the small satellite droplets due to limitations in imaging resolution. We investigated three liquid–liquid systems: aqueous ferrofluid/oil, aqueous ferrofluid/oil with the anionic surfactant sodium dodecyl sulfate (SDS), and aqueous ferrofluid/oil with the nonionic surfactant pentaethylene glycol monododecyl ether (C_12_E_5_). In addition to PS substrates, we also tested two superhydrophobic surfaces: a copper surface coated with nanorough silver and fluorinated thiol, and a glass slide coated with the commercial coating Glaco. Despite the differences in satellite droplet formation dynamics on different surfaces, we did not observe any change in the number of split daughter droplets in a given magnetic field. This is further discussed in the Supporting Information, while the experiments described here were performed in PS containers.

To investigate the effect of ferrofluid magnetization and interfacial tension systematically, we varied *c*_SPION_ from 8 to 25 vol% (corresponding to ≈0.8–2.4 mmol L^−1^ of SPIONs, calculated from the size distribution of the nanoparticles)^[^ ^22^ ^]^ and surfactant concentrations *c* from 0 to 17 mmol L^−1^ (**Figure** **3**a,b). At a low *c*_SPION_ of 8 vol% with no surfactant, *B*_e_ dominates over *S* leading to flattened droplets, and splitting does not occur (Figure S4, Supporting Information). Increasing *c*_SPION_ to 12 vol% results in four splitting events, but the resulting droplets are still flattened due to *f*_N_, leading to a labyrinthine pattern. Further increasing *c*_SPION_ to 17 vol% results in discrete, conical droplets. On the other hand, addition of surfactant (SDS) allows droplet splitting even with a low *c*_SPION_ of 8 vol%. However, the droplets adopt a dumbbell‐like shape due to droplet flattening. For high *c*_SPION_ (17 vol%), increasing the SDS concentration *c*_SDS_ from 0 to 17 mmol L^−1^ leads to almost a sevenfold increase in the number of split, conical droplets. Nonionic surfactant C_12_E_5_ allows reaching very low IFT values without adding a co‐surfactant or salt in the system (Figures S5 and S6, Supporting Information).^[^ ^34^ ^]^ This not only leads to smaller, but also deformed droplets (Figure 3b), as the droplets become more and more elongated in the lateral direction. At *c*_C12E5_ = 17 mmol L^−1^, ribbons and complex shapes are created instead of well‐defined droplets with narrow size distribution (Figure 3b, top image).

**Figure 3:**
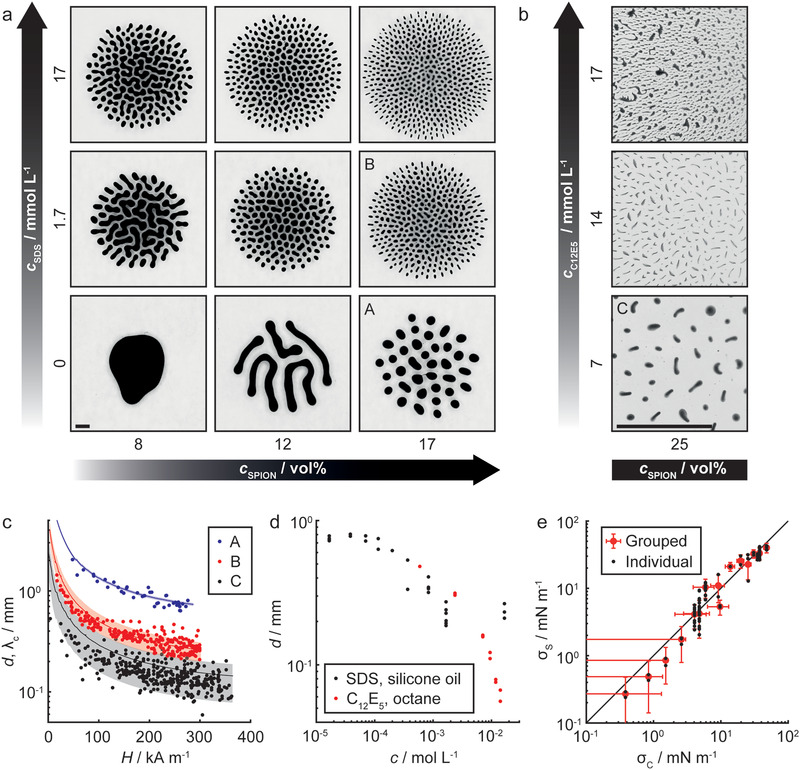
Droplet populations. a) Droplet populations created by field‐induced splitting for different SDS and SPION concentrations (*c*_SDS_ and *c*_SPION_) in silicone oil. Initial droplet volume *V*_0_ = 5 µL and external magnetic field *H* = 290 kA m^−1^. SDS lowers the IFT between ferrofluid and silicone oil, leading to smaller droplets. Low SPION concentration leads to dumbbell‐shaped droplets and labyrinthine patterns, whereas high concentration allows formation of distinct droplets. Scale bar: 1 mm. b) Split ferrofluid droplets in octane with different concentrations of C_12_E_5_ *c*_C12E5_(*V*_0_ = 0.2 µL, *H* = 300 kA m^−1^). C_12_E_5_ lowers IFT, leading to droplets with elongated cross sections. At 17 mmol L^−1^, ribbons are formed in addition to irregular droplets (top photo). Scale bar: 1 mm. c) Theoretically calculated critical wavelengths *λ*_c_ (lines) and droplet cross‐sectional major axes *d* (dots) for experimentally observed splitting events as a function of *H*. Shaded area represents uncertainty of the theoretical prediction (±1 standard deviation). A) 17 vol% SPIONs (droplet population shown in panel (a)); B) 17 vol% SPIONs, 1.7 mmol L^−1^ SDS (panel (a)); and C) 25 vol% SPIONs, 7.1 mmol L^−1^ C_12_E_5_ (panel (b)). d) *d* as a function of surfactant concentration *c* (17–25 vol% SPIONs, normal force density *f*_N_ = 2 MN m^−3^). e) IFT measured using splitting experiments *σ*_S_ as a function of IFT measured with control methods *σ*_C_ (pendant droplet and micropipette aspiration). The solid line has a slope of one. Black dots: individual experiments; red circles: experiments grouped based on control method IFT (*n* = 2–23). Error bars represent uncertainty (±1 standard deviation).

The simple approximation of the critical wavelength (Equation (1)) holds well for a wide range of magnetic field strengths as well as SPION and surfactant concentrations. As an example, Figure 3c presents experimental cross‐sectional major axes *d* of unstable droplets (dots) and theoretically calculated critical wavelengths *λ*_c_ (solid curves) as a function of external magnetic field *H*. The shaded area corresponds to the uncertainty of the theoretical prediction (±1 standard deviation) arising from the uncertainty of the IFTs measured with pendant droplet and micropipette aspiration techniques (see the Supporting Information for more details on these measurements). The theory holds for a system A) without surfactant, B) with SDS, and C) with C_12_E_5_. However, Equation (1) does not describe the appearance of dumbbell‐shaped droplets or labyrinthine patterns, which are typical for confined films of ferrofluids.^[^ ^1^ ^]^ In our experiments, the ferrofluid is not mechanically confined, and we instead hypothesize that *f*_N_ is sufficiently strong to cause a similar effect.

An increase in surfactant concentration leads to a decrease in *d* as predicted by Equation (1), as shown in Figure 3d for droplets affected by *f*_N_ = 2 MN m^−3^. As mentioned earlier, C_12_E_5_ allows for the creation of smaller droplets (*d* ≈ 50 µm) than SDS (*d* ≈ 200 µm). With SDS *d* plateaus for *c*_SDS_ > 1.7 mmol L^−1^, but becomes ill‐defined with C_12_E_5_, for *c*_C12E5_ > 17 mmol L^−1^, as the droplet shapes and sizes are no longer uniform. After this limit, the theory presented by Equation (1) is no longer sufficient to describe the system. This limit corresponds to an IFT of ≈0.1 mN m^−1^. For higher IFTs, Equation (1) holds and *d* can be used to determine the interfacial tension between the ferrofluid and the surrounding liquid, when the magnetization and field properties are known. This is demonstrated in Figure 3e, where the IFT as measured using splitting experiments is compared to values measured with micropipette aspiration (verified with the pendant droplet method for IFTs > 3 mN m^−1^). The method presented here can also be employed by simply calculating the number of droplets at different magnetic field strengths, if the shape of each droplet is assumed identical, making the technique optically less demanding. This is further described in the Supporting Information (Figure S7, Supporting Information). It is important to note that SPIONs themselves also affect the IFT (Figure S6, Supporting Information), which needs to be taken into account if the method is used to quantify surfactant concentrations, for example.

Understanding the role of SPION concentration and interfacial tension in field‐induced splitting allows for the creation of self‐assembled droplet populations in a controlled manner. These could serve as a platform for a new kind of population‐based digital microfluidics. Droplet division is difficult to achieve in conventional magnetic digital microfluidics without irreversibly pinning the droplet,^[^ ^10^ ^]^ but is easy using the field‐induced droplet splitting. Droplet combination is possible by rotating the magnet by 90°, which induces horizontal magnetization and mutual attraction between the droplets (**Figure** **4**a,b; Movie S3, Supporting Information). The self‐assembled population can be transported as a whole with a single vertical magnet, while keeping the droplets separate due to their mutual repulsion. The lubricating layer of immiscible liquid prevents pinning and allows moving the droplets with little friction (Figure S2, and Movies S4 and S5, Supporting Information). Individual droplets can be extracted from the population as needed with the help of another magnet (Figure 4c,d; Movie S6, Supporting Information). Thus, it is possible to switch between traditional droplet‐based microfluidics scheme, where all the droplets are addressed simultaneously via flow control, and digital microfluidics scheme, where droplets are addressed individually. Combining these concepts would allow developing more flexible droplet manipulation solutions. Since the population‐based digital microfluidics concept is based on permanent magnets, manual devices working without electricity could also be designed for field operations in remote locations.

**Figure 4:**
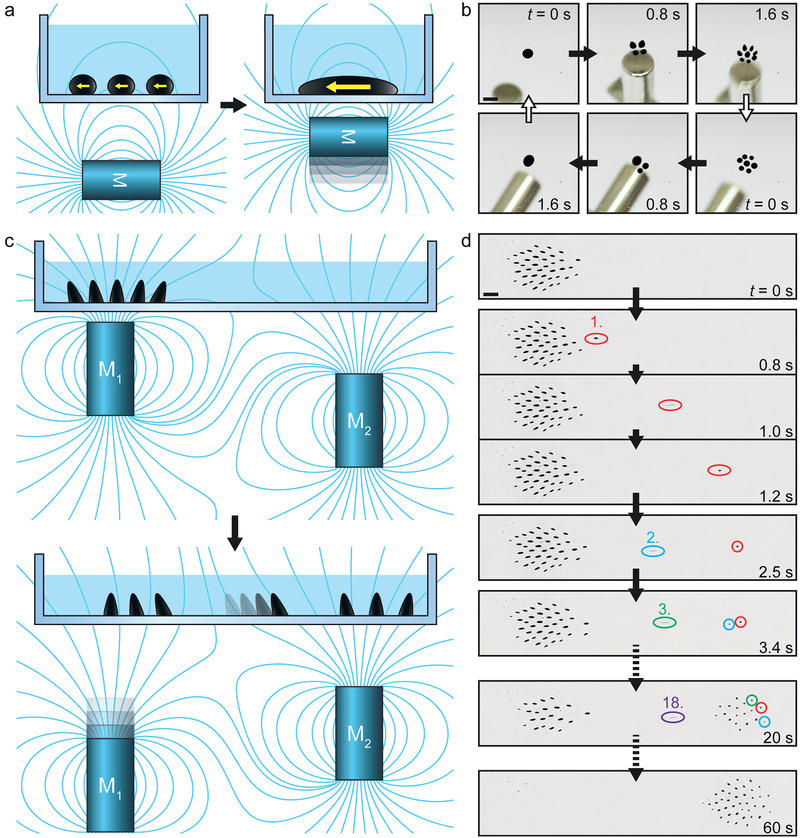
Microfluidics operations. a) Schematic of field‐induced droplet combination. As a horizontally oriented magnet is brought closer, the ferrofluid droplets magnetize horizontally (yellow arrows) and combine due to their mutual attraction. b) Image series of splitting (top row) and combining (bottom row) a ferrofluid droplet with a magnetic field (Movie S3, Supporting Information). Scale bar: 2 mm. c) Schematic of sequential transport of droplets between populations. As magnet M_1_ is lowered away from the droplets, they are increasingly pulled toward M_2_ due to the magnetic field (cyan lines), until they slide one by one from above M_1_ to above M_2_. d) Top‐view image series of sequential transport of ferrofluid droplets (numbered in the order of movement) with two magnets (Movie S6, Supporting Information). Scale bar: 1 mm.

In this work, we investigated magnetic‐field‐induced splitting of aqueous ferrofluid microdroplets immersed in an immiscible liquid. The formation of satellite droplets during the splitting process was studied using high‐speed imaging, and while the surface roughness affected splitting dynamics, it did not have a strong effect on the final droplet size and number. The mother droplet stability was shown to follow a simple theory (Equation (1)) over a wide range of interfacial tension and magnetization values, which were investigated by varying surfactant and magnetic nanoparticle concentrations. Self‐assembled droplet populations created by sequential splitting events were systematically studied, and regimes of labyrinthine patterns, dumbbell shaped, and conical droplets were identified. These results can be used to develop methods for measuring interfacial tension in liquid–liquid systems as well as novel digital microfluidics concepts using magnetically controlled ferrofluid droplet populations.

## Experimental Section

##### Splitting Experiments

A typical splitting experiment was performed as follows: a PS Petri dish (10 cm diameter, VWR) or a transparent, flat‐sided container (25 × 25 × 16 mm^3^, Ted Pella, Inc.) was filled with ≈3 mL of 5 cSt silicone oil or octane. Low interfacial tension experiments were done by adding either SDS to the ferrofluid or C_12_E_5_ to the outer phase (octane). All chemicals were purchased from Sigma–Aldrich. A neodymium magnet (diameter = 20 mm, height = 42 mm; Supermagnete) was attached to a computer‐controlled linear stage (Zaber X‐LSQ300B) underneath the container. At the beginning of the experiment, the magnet was far away (>100 mm) from the container, creating a field of ≈1.2 kA m^−1^ at the container bottom. A ferrofluid droplet (0.5–5 µL when using SDS, 0.2 µL when using C_12_E_5_) was pipetted in the filled container. To avoid the effect of electrostatic charging on the droplet, an electrostatic gate was passed around the sample. The droplet was left to equilibrate for 3 min, after which the magnet was lifted toward the ferrofluid at a velocity of 1 mm s^−1^ until a minimum distance (2–5 mm, creating a field of 280–410 kA m^−1^ at the container bottom) was reached. The increasing magnetic field and gradient induced droplet splitting, which was captured by recording a video with a digital single‐lens reflex camera (Canon EOS 60D). The videos were analyzed with custom Matlab functions to extract droplet positions and cross sections, which were fitted with ellipses. The beginning of an individual splitting event was identified by a decrease in minor axis length of the droplet cross section (Movie S7, Supporting Information). The corresponding major axis length was used as the experimental critical wavelength.

##### High‐Speed Imaging

Top‐ and side‐view high‐speed imaging were performed using two synchronized high‐speed cameras (Phantom Miro M310 and Phantom v1610) at a frame rate of 11 200 fps using two macrolenses (with a resolution of ≈4.3 µm pixel^−1^). To make the side‐view imaging easier to analyze, a rectangular magnet (100 × 13 × 6 mm^3^) was used, which induces splitting along the direction of the longest magnet side. More information can be found in the Supporting information.

##### Statistical Analysis

In splitting experiments, video data were preprocessed with automatic thresholding; droplets were resolved with image recognition; and incorrectly identified droplets were removed using custom Matlab functions. All data were presented as mean ± standard deviation. In grouped splitting experiments presented in Figure 3c, sample size *n* = 2–23. All statistical analyses were calculated with Matlab.

## Conflict of Interest

The authors declare no conflict of interest.

## Supporting information

Supporting Information

Supplemental Movie 1

Supplemental Movie 2

Supplemental Movie 3

Supplemental Movie 4

Supplemental Movie 5

Supplemental Movie 6

Supplemental Movie 7
