# Supplementary material for: Ferrofluid Microdroplet Splitting for Population‐Based Microfluidics and Interfacial Tensiometry
Source: Adv Sci (Weinh). 2020 Jun 9;7(14):2000359. doi: 10.1002/advs.202000359 (PMC7375242; doi:10.1002/advs.202000359)
Supplement: Supplementary file 1 — Supporting Information [file ADVS-7-2000359-s001.pdf]

Copyright WILEY-VCH Verlag GmbH & Co. KGaA, 69469 Weinheim, Germany, 2020.

Supporting Information

Ferrofluid microdroplet splitting for population-based microfluidics and interfacial tensiometry

Mika Latikka, Matilda Backholm, Avijit Baidya, Alberto Ballesio, Amandine Serve, Gregory Beaune, Jaakko Timonen, Thalappil Pradeep and Robin Ras*

Supplementary movies

Movie S1: Magnetic field induced splitting. A) 5 µL aqueous ferrofluid droplet with SDS in silicone oil (top view analysis). B) 1 µL aqueous ferrofluid droplet in octane (top and side views).

Movie S2: High-speed videos of a field-induced splitting of a 5 µL ferrofluid droplet in 5 cSt silicone oil on a Glaco-coated substrate. The videos were recorded at 11200 fps.

Movie S3: Sequential splitting and combination of 1.5 µL ferrofluid droplet in 5 cSt silicone oil with a small cylindrical magnet (diameter 4.5, length 9 mm).

Movie S4: Ferrofluid droplet population (initial volume 5 µL) immersed in 5 cSt silicone oil moved around on a polystyrene surface with a permanent magnet.

Movie S5: Ferrofluid droplet population (initial volume 5 µL) in air moved around on a polystyrene surface with a permanent magnet. The droplets pin to the surface, leaving behind small sessile droplets.

Movie S6: Sequential droplet transport from one ferrofluid droplet population to another with two magnets in 5 cSt silicone oil.

Movie S7: Analysis of magnetic field induced splitting of a 2 µL ferrofluid droplet in octane. When a ferrofluid droplet becomes unstable, its minor axis (top view) starts to decrease, and corresponding major axis is the maximum droplet diameter.

Properties of the permanent magnets and the ferrofluids

Magnets

Most of the splitting experiments were performed using a cylindrical neodymium permanent magnet (diameter 20 mm, height 42 mm, Supermagnete, **Figure S1**a). Investigation of the satellite droplet formation using high-speed imaging was done with a rectangular magnet (100 x 13 x 6 mm^3, K&J Magnetics), which induces splitting along the direction of the longest magnet side. Predictable splitting direction made the side view imaging easier.
During data analysis the magnetic field affecting the droplets was evaluated based on the field profile of the magnet measured with a gaussmeter (Lakeshore 410), magnet's vertical distance from the ferrofluid and droplet shapes. Droplets' horizontal distance from the magnet axis was assumed small, and the field value was calculated at the magnet axis. The field was calculated at the approximate height of the mass center of the droplets, which was estimated based on total ferrofluid volume and cross-sectional area of the droplets assuming the droplet shape to be half of an ellipsoid. Demagnetizing field was calculated assuming ellipsoidal droplets.[1]

Ferrofluid synthesis

Aqueous ferrofluid was synthesized using the co-precipitation method[2] and stabilized with citric acid near pH 7, as described earlier.[3] The excess water was evaporated in room temperature until the ferrofluid contained up to 25 vol% of nanoparticles. Ferrofluid was diluted with ultrapure water for the experiments as needed. Approximate superparamagnetic iron oxide nanoparticle (SPION) volume percent c_{SPION} is calculated from ferrofluid density assuming that ferrofluid consists only of water and SPIONs (SPION density is assumed 5175 kg m^{-3}). According to XPS and FTIR spectra the nanoparticles consist of magnetite.[3]

Ferrofluid characterization

Ferrofluid samples (7 µl) were sealed in polypropylene powder cups with vacuum grease and Parafilm. Hysteresis loops were measured with vibrating sample magnetometer (Quantum Design PPMS Dynacool) using field values of -9 – 9 T (Figure S1b-d).

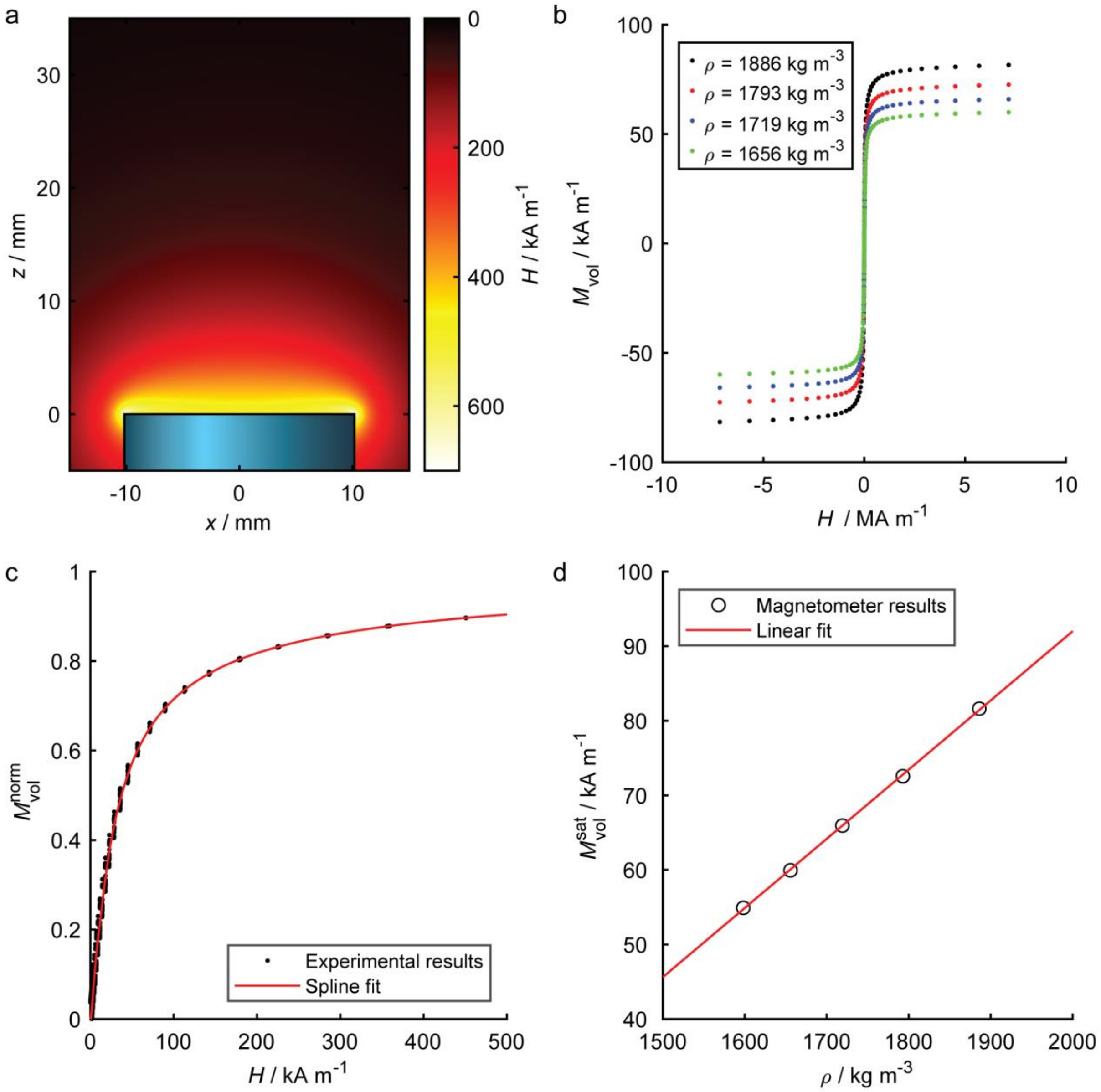

Figure S1: Magnetic properties of the permanent magnet and the ferrofluids. a) Magnetic field H created by the cylindrical permanent magnet used in most of the splitting experiments. b) Magnetic hysteresis loops with different ferrofluid densities. c) Normalized magnetization $M_{\text{vol}}^{\text{norm}} = M_{\text{vol}}/M_{\text{vol}}^{\text{sat}}$ as a function of applied magnetic field. d) Saturation magnetization $M_{\text{vol}}^{\text{sat}}$ as a function of ferrofluid density ρ.

Low-friction surfaces

In order to achieve individual, highly mobile droplets via magnetic field induced splitting, the surface supporting the droplets has to be sufficiently repellent to the aqueous ferrofluid. This can be achieved by using a superhydrophobic surface[4] or a lubricating layer of immiscible liquid (**Figure S2**). In case of lubricated surface the splitting dynamics depend on the surface roughness. On a rough surface (Figure S2c) the increased flow in the roughness reduces the viscous dissipation when the droplet moves compared to a flat surface (Figure S2b), which leads to faster splitting dynamics (**Figure S3**).
When ferrofluid droplets in air are actuated on a smooth polystyrene (PS) surface by horizontally moving the permanent magnet below the substrate, the droplets pin to the surface, leaving behind small immobile droplets (Figure S3d, **Movie S5**). However, when the droplets are immersed in silicone oil, they can be moved without pinning (Figure S3e, **Movie S4**).

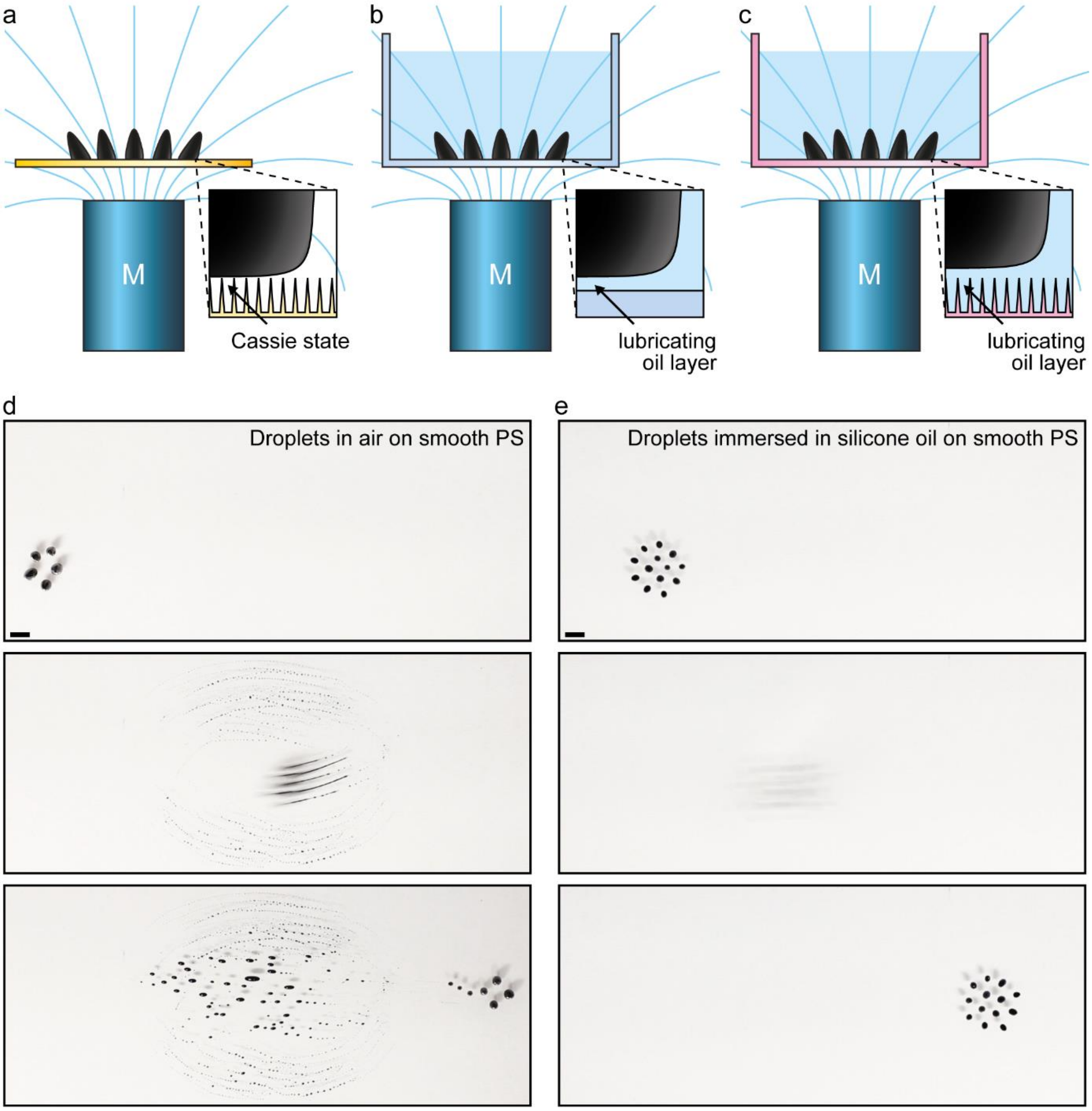

Figure S2: Schematic of droplet populations on different low-friction water-repellent surfaces. Adhesion between the droplets and the surface is reduced by the Cassie state on a superhydrophobic surface (a) and a lubricating oil layer for droplets immersed in immiscible liquid (b and c). Splitting dynamics are slower on flat lubricated surfaces (b) compared to rough lubricated surfaces (c). Magnetic field lines created by the permanent magnet below the droplets are shown in cyan. d) Droplet population (initial ferrofluid volume 5 μL) in air moved around on a PS surface with a permanent magnet below the substrate. The droplets pin to the surface during transport, leaving behind small sessile droplets (Movie S5). e) As (d), but the droplets are immersed in 5 cSt silicone oil, which prevents pinning to the surface (Movie S4). Scale bars 2 mm.

Field-induced splitting on different surfaces

Atomic force microscopy

The effect of surface roughness on splitting dynamics was investigated using a PS surface and a PS surface coated with the commercial superhydrophobic spray Glaco. To assess the roughness of the two surfaces, atomic force microscopy (AFM) imaging was performed in air by using Dimension icon (Bruker instrument). The images were acquired in ScanAsyst Peak force tapping mode by using rectangular shaped silicon cantilevers (OTESPA-R3 Bruker) with a nominal spring constant of 26 N m^{-1}, resonance frequency in air 300 KHz and typical tip curvature radius of 8 nm. Images of (50 × 50 μm^2) were collected with 256 data points per line at the rate of 1 Hz with the peak force amplitude of 150 nm. RMS roughness values were 6.2 ± 0.2 nm for PS and 43 ± 3 nm for Glaco-coated PS surface (Figure S3a-b**)**. The values are mean and standard deviations of three measurements on different parts of the samples.

High-speed imaging on different surfaces

Satellite droplet formation on the two surfaces was investigated using high-speed imaging. At the beginning of the experiment, the rectangular magnet was located 8 cm below the container to keep the magnetic field affecting the droplets small. The magnet was then moved up with a motorized stage at a speed of 1 mm s^{-1} until it almost touched the container. The experiments were performed with a 5 μL ferrofluid mother droplet with SPION volume percent c_{SPION} = 22 – 25 vol%, which corresponds to ferrofluid densities ρ = 1.938 – 2.060 g mL^{-1} (Figure S3c-d). Lower SPION concentrations could not be investigated, as the magnet was not powerful enough to split a 5 μL droplet of less concentrated ferrofluid.

When viewed from the top the breakup process looks very similar to the breakup of a viscous, Newtonian capillary bridge in a viscous, infinite medium,[5] where a thickening of the middle of the bridge can be seen at early times (**Figure 2**b). As the largest satellite droplet is being formed in the center, the narrow side bridges protruding from its sides evolve and break up in a repeated, self-similar fashion into several tiny subsatellite droplets. Interestingly, the W-shape of the time evolution on the Glaco-coated substrate is qualitatively very similar to what was found in the work by Tjahjadi et al.,[5] where the breakup first started at the edges of the bridge and then continued from the center after a short time. The pure PS substrate seems to render stronger drag between the ferrofluid and the solid substrate, leading to slower breakup dynamics that, furthermore, proceeds in the opposite direction as compared to rougher Glaco-coated substrate.

Splitting-based IFT measurements on different surfaces

We used three different substrates to measure interfacial tension (IFT) between the ferrofluid and 5 cSt silicone oil. No significant difference in the measured IFT was observed between different substrates: a PS container (IFT 33 ± 7 mN m^{-1}), a glass surface coated with Glaco (IFT 32 ± 8 mN m^{-1}) and a copper substrate coated with nanorough silver and fluorinated thiol to make it superhydrophobic (IFT 30 ± 8 mN m^{-1}).[6] Pendant droplet method was used as a control technique, and results obtained with splitting experiments agree with it within experimental accuracy (IFT 38 ± 2 mN m^{-1}). Uncertainties represent standard deviation.

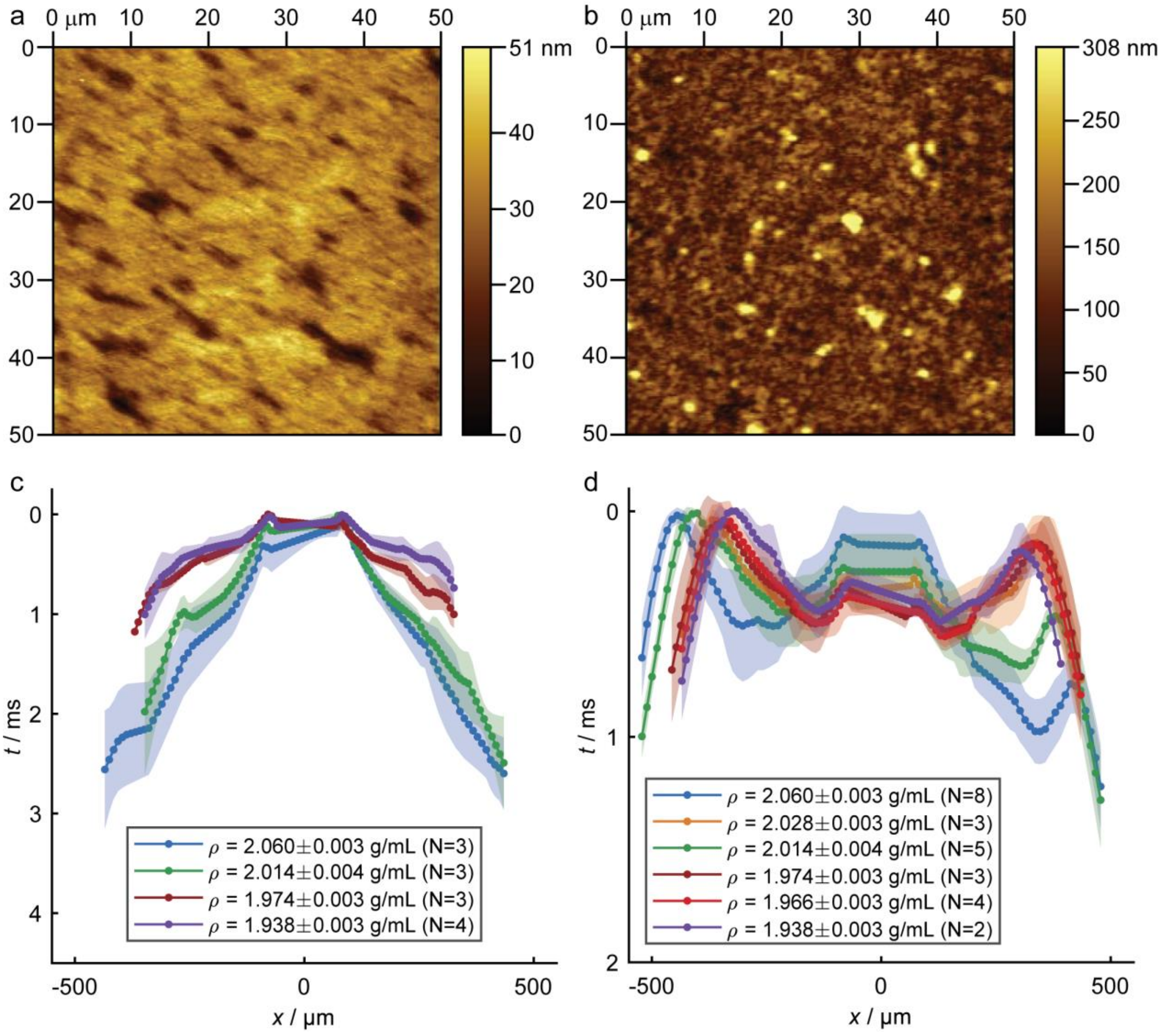

Figure S3: Satellite droplet formation dynamics on different surfaces. a-b) AFM measurements of a) PS container and b) Glaco-coated PS container. c-d) Averaged time evolution of the satellite droplet formation dynamics on c) PS container and d) Glaco-coated PS container for different ferrofluid densities ρ. The shaded area shows the standard deviation of multiple (N) experiments.

Ferrofluid droplet shape in a magnetic field

As the cylindrical permanent magnet underneath the ferrofluid is brought closer, magnetic field and vertical field gradient are increased. However, the field gradient increases more rapidly than the field strength and magnetization (**Figure S4**a). Consequently, as the magnet is brought closer to the ferrofluid the effective Bond number B_e, which is related to droplet flattening, increases compared to the dimensionless number S, which is related to droplet elongation. As a result, the droplets are typically first elongated and then flattened during splitting experiment. The droplet shape depends also on the SPION concentration and IFT (Figure S4b-d).

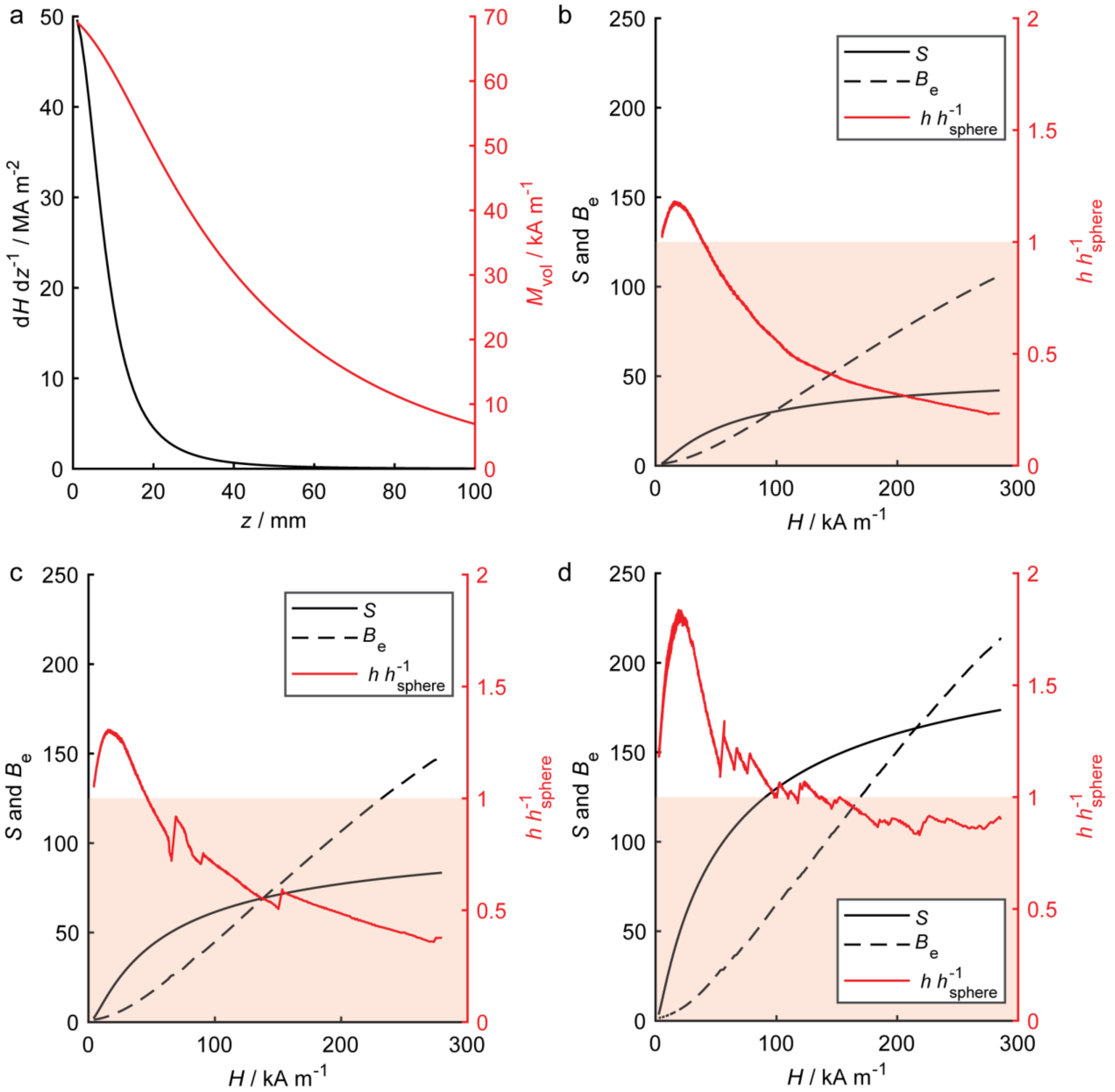

Figure S4: Ferrofluid droplet shape in magnetic field. a) Field gradient dH dz^{-1} and ferrofluid magnetization M_{vol} (c_{SPION} = 20 vol%, with no demagnetizing field) as a function of the vertical distance z from the surface of the cylindrical magnet. dH dz^{-1} grows more rapidly than M_{vol} when z is small. b-d) show the relative magnitudes of S and B_e, and the height of the droplets h compared to a height of a sphere with equal volume h_{sphere}. Red shaded area corresponds to flattened droplets. h is calculated from the top view images using the total ferrofluid volume and cross-sectional droplet areas by assuming ellipsoidal droplet shape. As S, B_e and h are approximations, only a qualitative agreement is expected. b) With c_{SPION} = 8 vol% B_e dominates S already at relatively small magnetic fields and droplet is flattened. c) Higher c_{SPION} (12 vol%) leads to a smaller difference between B_e and S, and less flattened droplets. d) With c_{SPION} = 17 vol% S is significantly larger than B_e and droplets are elongated at low magnetic fields. At high fields B_e is approximately equal to S, and the droplet heights remains close to that of corresponding spheres.

Droplet self-assembly

Ferrofluid droplets self-assemble due to their attraction to the magnet axis and their mutual dipolar repulsion. The magnetic field H_{mag} created by the permanent magnet near the magnet axis is approximately parabolic:[4]

$$H_{\mathrm{mag}} = H_0(z) - \frac{1}{2}cl^2$$

where H_0 is the field at the magnet axis at distance z from magnet surface, $c = -d^2H/dl^2$ is the radial field curvature and l is distance from the magnet axis.
Magnetized ferrofluid droplet creates a dipolar field:[7]

$$H_{\mathrm{drop}}(\bar{r}) = \frac{1}{4\pi}\left(\frac{3\bar{r}(\bar{m}\cdot\bar{r})}{|\bar{r}|^5} - \frac{\bar{m}}{|\bar{r}|^3}\right)$$

where *r* is the location vector and *m* is the magnetic dipole moment of the droplet. The energy *U* of a magnetic dipole in a magnetic field *H* is:[7]

$$U = -\mu_0\bar{m}\cdot\bar{H}$$

The droplets are confined to (x,y)-plane, while the dipolar moments m_i are approximately parallel to z-direction, and as a result $\bar{m}_i \cdot (\bar{r}_i - \bar{r}_j) \approx 0$ and $\bar{m}_i \cdot \bar{m}_j \approx m_i m_j$. The interdroplet dipolar interaction energy U_{dipolar} of the droplet population is then:

$$U_{\mathrm{dipolar}} = \frac{\mu_0}{4\pi}\sum_{i=1}^{N}\sum_{j=i+1}^{N}\frac{m_i m_j}{|\bar{r}_i - \bar{r}_j|^3}$$

The potential energy increases as the distance $|\bar{r}_i - \bar{r}_j|$ between the droplet decreases, leading to interdroplet repulsion.
The total energy U of the droplet population in the magnetic field created by the permanent magnet is:[4]

$$U = \frac{\mu_0}{4\pi}\sum_{i=1}^{N}\sum_{j=i+1}^{N}\frac{m_i m_j}{|\bar{r}_i - \bar{r}_j|^3} - \mu_0\sum_{i=1}^{N} m_i\left(H_0 - \frac{1}{2}c|r_i|^2\right)$$

Interfacial tension measurements

Pendant droplet measurements

IFTs between ferrofluid and surrounding media were measured with the pendant droplet method using Attension Theta tensiometer (Biolin Scientific). IFT value was recorded after equilibration time of 3 minutes, as with splitting experiments. Measurements were repeated using water instead of ferrofluid for comparison (**Figure S5**).

Micropipette aspiration measurements

Micropipettes were prepared by pulling borosilicate capillaries (WPI, 1 mm, 0.58 mm o.d./i.d.) using a flaming/brown type puller (P-97, Sutter Instrument Company). Afterward, the pipettes were sized to about 20 μm in inner diameter by using a microforge (MF-900, Narishige). To introduce pipettes horizontally into sample holders, they were bent by heating the pipette on a flame. Micropipettes were made hydrophobic by incubation for at least 1 h in an organic solution made of dichlorodimethylsilane (Sigma-Aldrich) diluted 20 times in cyclohexane. Pipettes were then filled with octane and connected to an octane reservoir attached to a piezoelectric pressure controller unit (Elveflow). The pressure was controlled with a precision of about 10 microbars using the instrument software during the measurements. Aspirated droplets of ferrofluid were visualized with an inverted microscope (Nikon Eclipse Ti) equipped with various objectives (from 1× to 60×) associated with a 1.5× magnification device. Droplets inside micropipettes were observed using a CCD camera (Andor Zyla Scmos). All experiments were performed at ambient pressure using an in-house built observation chamber. A 1-10 µL pipette tip connected to the corresponding pipette was filled with successive "layers" of the octane – surfactant solution, ferrofluid, air and ferrofluid (**Figure S6**a). This strategy was used in order to limit the evaporation of the solvents during the experiments. The tip was then removed from the pipette and cut with a razor. The tip, which was our observation chamber, was placed on a microscopy slide on the stage of the microscope and the micropipette was inserted in the octane phase from the larger aperture. A pressure P_0 was applied by the pressure controller to compensate for the high between the octane tank and the sample. There was no flow in the observation chamber when ΔP_0 was applied. The micropipette was put in contact with the ferrofluid and a pressure $\Delta P > \Delta P_0$ was applied to aspirate the ferrofluid in the micropipette (Figure S6b). The pressure was decreased until the penetration length of the ferrofluid inside the pipette was equal to the radius of the micropipette R_p. This pressure was called the critical pressure ΔP_c. If we continued to decrease the pressure, a large volume of ferrofluid was aspirated in the micropipette.

Knowing ΔP_c, the interfacial tension σ between the ferrofluid and octane – surfactant solution could be determine using the Laplace law $\Delta P_c = 2\sigma\left(\frac{1}{R_p} - \frac{1}{R_0}\right)$, where R_0 is the radius of curvature of the ferrofluid – octane interface.

Three sets of experiments were performed using octane solutions containing different concentrations of $C_{12}E_5$ surfactant (0, 0.6, 2, 5, 7, 9, 12 and 14 mmol L^{-1}) (Figure S6c).

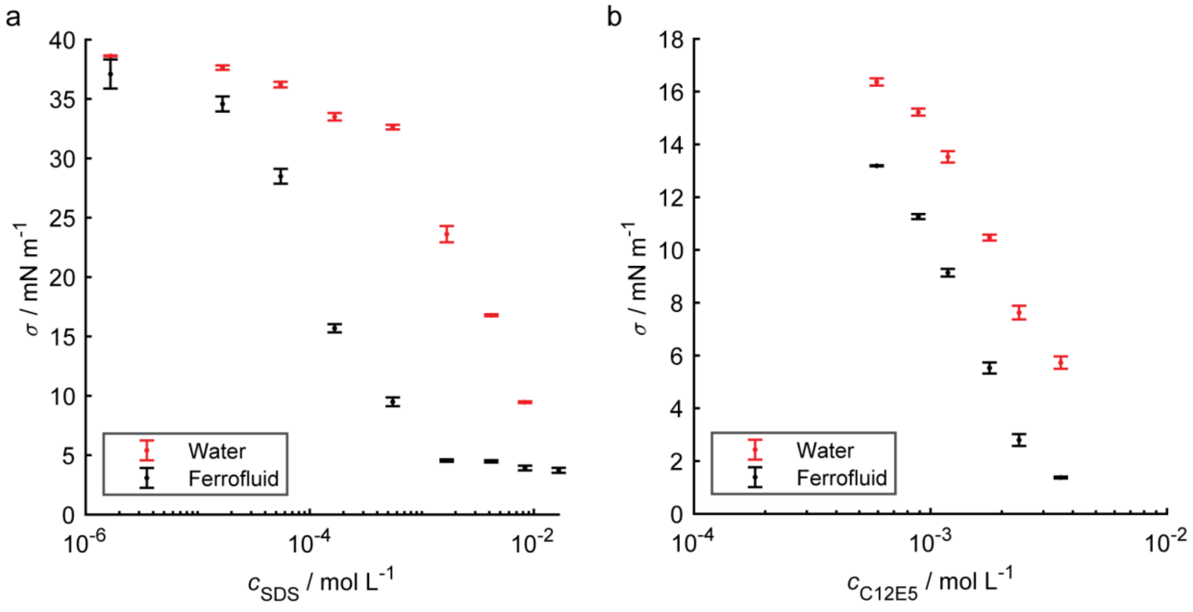

Figure S5: Effect of SPIONs to IFT. IFT was measured for one minute after equilibration time of three minutes. The values are mean values of 2 – 5 measurements and error bars represent standard deviation. a) IFT of water and ferrofluid droplets in silicone oil with sodium dodecyl sulfate (SDS) as a function of SDS concentration c_{SDS}. b) IFT of water and ferrofluid droplets with pentaethylene glycol monododecyl ether ($C_{12}E_5$) in octane as a function of $C_{12}E_5$ concentration c_{C12E5}.

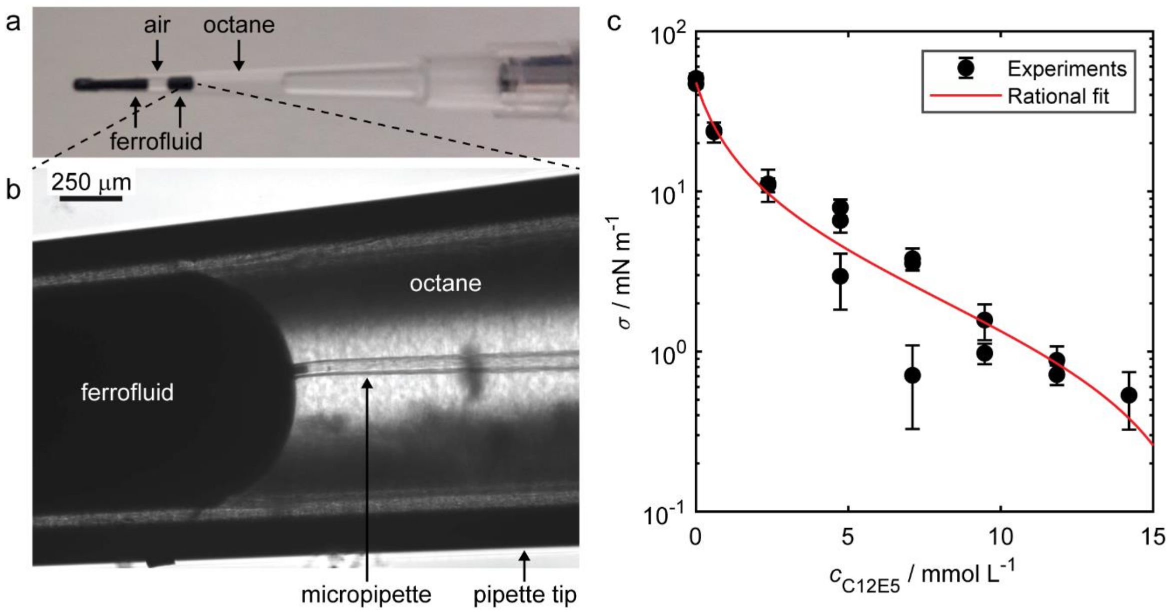

Figure S6: Micropipette aspiration measurements. a) Typical pipette tip in which the micropipette experiments were performed. b) Aspiration of the ferrofluid inside a micropipette at the ferrofluid/octane interface. c) IFT of ferrofluid in octane with $C_{12}E_5$ as a function of c_{C12E5}. The black circles are mean values of 3 to 10 measurements and error bars represent standard deviation. The data was fitted with a rational function $(p_1\,x + p_2)/(x + q_1)$ (red line) weighted by the inverse of standard deviations of the data

points. The two outlying data points with lower IFT (c_{C12E5} = 4.7 and 7.1 mmol L^{-1}) are probably due to evaporation of the octane during the experiments.

IFT measurements based on number of droplets

Instead of optically measuring the droplet diameters d as they split due to the field-induced instability, the critical wavelength λ_c can be approximated with the help of the total ferrofluid volume V_0 and assuming the droplets are identical half ellipsoids. Volume of a single droplet $V = V_0/n = 2/3\,\pi\,(d/2)^2 h$, where n is the number of droplets and h is the droplet height. Right before splitting event $\lambda_c = d = \sqrt[3]{6V_0/(\pi\;n\;a)}$, where $a = h\,d^{-1}$ is the droplet aspect ratio, which can be treated as a free parameter. When analyzing all the splitting experiments, a = 1.15 minimizes the cumulative relative errors compared to control IFT measurements using pendant droplet and micropipette aspiration techniques (**Figure S7**).

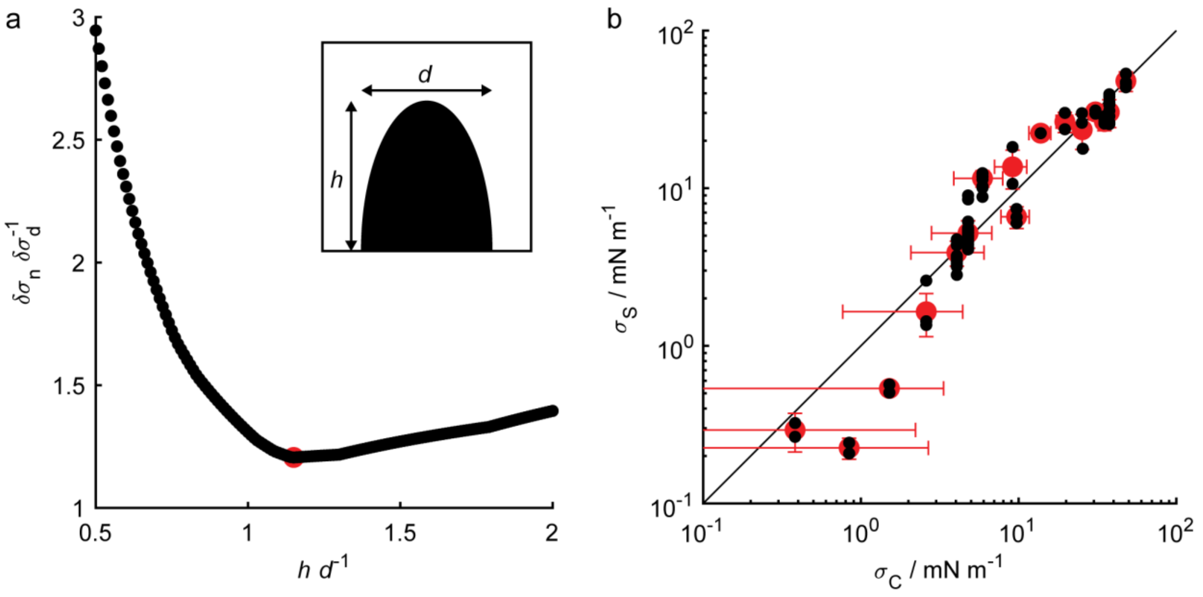

Figure S7: IFT measurement based on number of split droplets. a) Comparison of errors in measured IFT for different analysis methods: σ_n is based on number of split droplets and σ_d is based on droplet diameters. $\delta\sigma_n$ and $\delta\sigma_d$ are cumulative relative errors compared to control method $\delta\sigma_C$ (pendant droplet and micropipette aspiration) over all measurements: $\delta\sigma_n = \sum|\sigma_n - \sigma_C|/\sigma_C$. The relative error is plotted as a function of droplet aspect ratio $h\ d^{-1}$, where h is the height of the droplet and d is the diameter of droplet base. Droplet is assumed a half of an ellipsoid. Inset shows the droplet shape, which minimizes the error ($h\ d^{-1}$ = 1.15, red circle in the main figure). b) IFT based on number of droplets in splitting experiments σ_S as a function of IFT measured with control methods σ_C (pendant droplet and micropipette aspiration). Black dots: individual experiments, red circles: experiments grouped based on control method IFT. Error bars represent uncertainty (±1 standard deviation).

Image processing

Brightness and contrast of the photographs and image series in Figures 1-4 were adjusted with Photoshop. In addition, photographs in Figure 1d and 3a-b were converted to grayscale and white balance of Figure 4b was adjusted.

References

[1] T. Vieu, C. Walter, *J. Fluid Mech.* **2018**, *840*, 455.
[2] R. Massart, *IEEE Trans. Magn.* **1981**, *17*, 1247.
[3] C. Vasilescu, M. Latikka, K. D. Knudsen, V. M. Garamus, V. Socoliuc, R. Turcu, E. Tombácz, D. Susan-Resiga, R. H. A. Ras, L. Vékás, *Soft Matter* **2018**, *14*, 6648.
[4] J. V. I. Timonen, M. Latikka, L. Leibler, R. H. A. Ras, O. Ikkala, *Science* **2013**, *341*, 253.
[5] M. Tjahjadi, H. A. Stone, J. M. Ottino, *J. Fluid Mech.* **1992**, *243*, 297.
[6] I. A. Larmour, S. E. J. Bell, G. C. Saunders, *Angew. Chem. Int. Ed.* **2007**, *46*, 1710.
[7] J. M. D. Coey, *Magnetism and Magnetic Materials*, Cambridge University Press, **2009**.
